# Heart Rate Variability Biofeedback as a Preventive E-Mental Health Intervention for Stress, Anxiety and Depression: Evidence 2020–2025

**DOI:** 10.1192/j.eurpsy.2026.11348

**Published:** 2026-07-31

**Authors:** S. A. Nalba, L. Boronin

**Affiliations:** Department of Mental Health, Medical Psychology and Psychotherapy, Nicolae Testemițanu State University of Medicine and Pharmacy, Chisinau, Moldova, Republic of

## Abstract

**Introduction:**

Heart rate variability biofeedback (HRV-BF) trains breathing at the resonance frequency (~6 breaths/min) to enhance baroreflexes, vagal tone, and emotion regulation. HRV reflects autonomic flexibility and allostatic capacity and is increasingly proposed as a pragmatic digital biomarker of stress responsivity. With expanding mobile delivery, HRV-BF provides scalable, low-cost stress-management and is positioned as a selective and indicated preventive intervention in e-mental health.

**Objectives:**

To synthesise recent evidence on HRV-BF effectiveness in preventing stress, anxiety, and depressive symptoms in at-risk but non-clinical populations, and to assess its role in scalable e-mental health strategies.

**Methods:**

A narrative synthesis of systematic reviews, meta-analyses, and RCTs (2020–2025) was performed via PubMed, Scopus, and Web of Science. Eligible studies involved non-clinical or at-risk groups (e.g., students, healthcare workers, individuals with elevated stress), used validated scales such as PHQ, GAD-7, and PSS, and reported outcomes on depression, anxiety, stress, or resilience. Interventions ranged from brief multi-session programs to app-based training. Exclusions: clinical psychiatric diagnoses, time frame outside 2020–2025, non-peer-reviewed sources, and reports lacking validated outcomes.

**Results:**

Across students, healthcare professionals, and young adults—with emerging data in older adults—HRV-BF produced small-to-moderate reductions in depressive and anxiety symptoms, alongside lower perceived stress. Gains extended to resilience, emotion regulation, and subjective control, with physiological changes indicating improved vagal regulation. Mobile and telemedicine formats showed high feasibility, good adherence, and short-term improvements, integrating well into educational and workplace settings. App-based observational data linked HRV coherence to improved affective states. Early findings highlight potential synergies with wearables, digital biomarkers, and AI-driven feedback to personalise training and sustain engagement.

**Conclusions:**

HRV-BF demonstrates preventive potential for stress, anxiety, and depression across populations and formats, consistent with stepped-care and public-health priorities. Limitations include small samples, heterogeneous protocols, and short follow-up. Large multicentre trials with active controls are needed to test durability, dose–response, and standardisation, plus economic evaluations in care settings. Embedding HRV-BF into early-intervention and relapse-prevention frameworks could enhance healthcare resilience and reduce the long-term burden of common mental disorders.

**Keywords:**

heart rate variability, biofeedback, stress prevention, anxiety, depression, e-mental health, mobile health, digital biomarkers, preventive psychiatry

**Disclosure of Interest:**

None Declared

